# Primary intraosseous carcinoma in the pediatric and adolescent mandible

**DOI:** 10.1186/s12957-021-02465-2

**Published:** 2022-01-27

**Authors:** Hyun Jun Oh, Dong Whan Shin, Hye-Jung Yoon, Hoon Myoung, Soung Min Kim

**Affiliations:** 1 Department of Oral and Maxillofacial Surgery, Dental Research Institute, School of Dentistry, Seoul National University, 101 Daehak-ro, Jongno-gu, Seoul, 03080 Korea; 2 Department of Oral Pathology, Dental Research Institute, School of Dentistry, Seoul National University, Seoul, Korea

**Keywords:** Odontogenic tumor, Odontogenic cyst, Primary intraosseous carcinoma (PIOC), Pediatric and adolescent mandible, PRISMA guideline

## Abstract

**Background:**

Primary intraosseous carcinoma (PIOC) is a rare malignant odontogenic tumor that predominantly occurs in males older than 50 years. PIOC can be misdiagnosed as odontogenic cyst because it occasionally shows a well-defined border on radiography. In this study, related literatures of pediatric and adolescent PIOC cases were analyzed under strict PRISMA guidelines along with an adolescent case who was provisionally misdiagnosed as an odontogenic cyst.

**Methods:**

All case reports for PIOC published in English from 1966 to 2021 were collected. Cases under the age of 20 were classified as pediatric and adolescent populations in this study. A total of 12 pediatric and adolescent cases including 11 PIOCs from the literature and one new case of a 14-year-old female were analyzed. Clinical and radiographic features, diagnosis and treatment approaches, and prognosis were investigated.

**Results:**

Ages ranged from 4 to 18 years. The female to male ratio was 1.4:1. Seven cases occurred in the mandible. Swelling was observed in 11 patients. The radiologic borders were well-defined in six cases and corticated in four cases. Tooth displacement and root resorption were observed in four and six cases, respectively. The provisional diagnosis for seven patients was odontogenic cyst and enucleation was performed in six cases including the new case. During the follow-up period, local recurrence occurred in three patients. The pediatric and adolescent PIOC cases with local recurrence showed poor prognosis. The locally recurred lesion in the new case did not decrease in size despite concurrent chemo-radiation therapy.

**Conclusions:**

Three-dimensional imaging modalities and incisional biopsy with multiple specimens are necessary to rule out PIOC in the lesions with atypical radiographic findings. PIOC should be diagnosed differentially from odontogenic cyst even in pediatric and adolescent populations to properly manage the disease with poor prognosis.

## Background

Primary intraosseous carcinoma (PIOC) is a rare and infrequently reported malignant odontogenic tumor. In 2005, the World health organization (WHO) divided PIOC into three subcategories according to histogenesis [1]. However, in 2017, WHO reclassified it as a single entity after leaving out unsubstantiated references to histogenesis [2]. Approximately 260 cases have been reported [3, 4]. PIOC is more common in males and usually occurs in people 50 years and older [3, 5–11]. PIOC has occurred extremely rarely in pediatric and adolescent populations: prior to this case, only 11 cases had been reported in the English literature [12–22]. PIOC is misdiagnosed frequently as odontogenic cyst because it occasionally shows well-defined borders in panoramic view or on computed tomography (CT) [23–26]. In this study, a PIOC case of a 14-year-old female patient who was provisionally diagnosed as odontogenic cyst is discussed along with a literature review of pediatric and adolescent PIOC cases.

## Methods

### Literature search

**Fig. 1:**
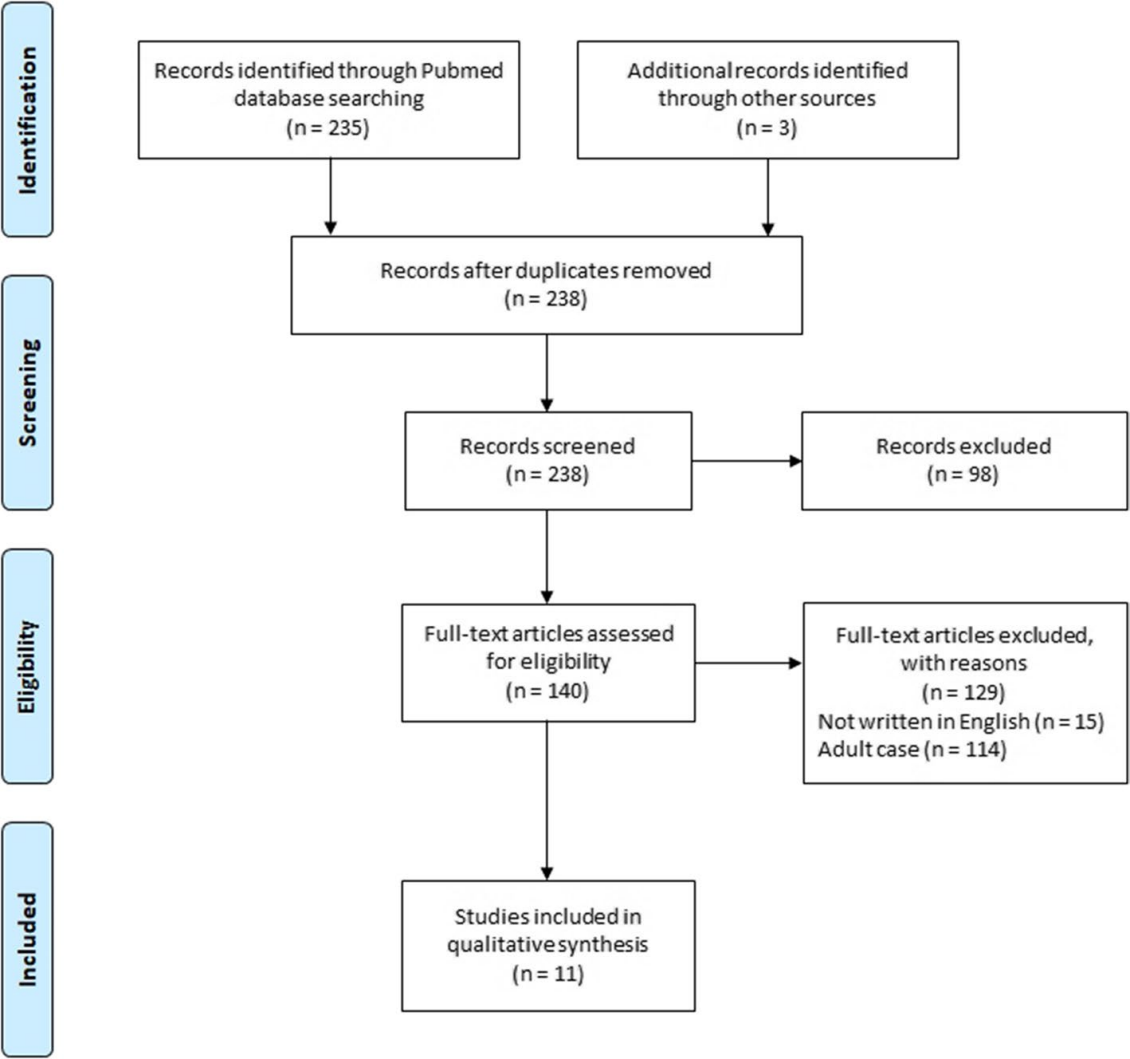
PRISMA flow chart of systematic search on PIOC. *PRISMA* Preferred Reporting Items for Systematic reviews and Meta-analyses, *PIOC* primary intraosseous carcinoma

This study followed the Preferred Reporting Items for Systematic Reviews and Meta-Analyses (PRISMA) guidelines [27] (Fig. 1). All case reports for PIOC published in English from 1966 to 2021 were collected. The search was carried out using the keywords “primary intraosseous carcinoma,” “primary intraosseous squamous cell carcinoma,” and “primary intra-alveolar carcinoma” in PubMed. The search string was ((“primaries”[All Fields] OR “primary”[All Fields]) AND (“intraosseal”[All Fields] OR “intraosseous”[All Fields] OR “intraosseously”[All Fields]) AND (“carcinoma”[MeSH Terms] OR “carcinoma”[All Fields] OR “carcinomas”[All Fields] OR “carcinoma s”[All Fields])) OR ((“primaries”[All Fields] OR “primary”[All Fields]) AND (“intraosseal”[All Fields] OR “intraosseous”[All Fields] OR “intraosseously”[All Fields]) AND (“carcinoma, squamous cell”[MeSH Terms] OR (“carcinoma”[All Fields] AND “squamous”[All Fields] AND “cell”[All Fields]) OR “squamous cell carcinoma”[All Fields] OR (“squamous”[All Fields] AND “cell”[All Fields] AND “carcinoma”[All Fields]))) OR ((“primaries”[All Fields] OR “primary”[All Fields]) AND “intra-alveolar”[All Fields] AND (“carcinoma”[MeSH Terms] OR “carcinoma”[All Fields] OR “carcinomas”[All Fields] OR “carcinoma s”[All Fields])). In addition, the reference lists of the retrieved articles were manually cross-checked.

### Eligibility criteria and data analysis

Publication reporting cases of PIOC of the maxilla or mandible were eligible. The inclusion criterion was PIOC cases under the age of 20. The exclusion criterion was articles not written in English. Clinical and radiographic characteristics, diagnosis and treatment approaches, and prognosis were analyzed.

### Case presentation of a 14-year-old Korean female

**Fig. 2:**
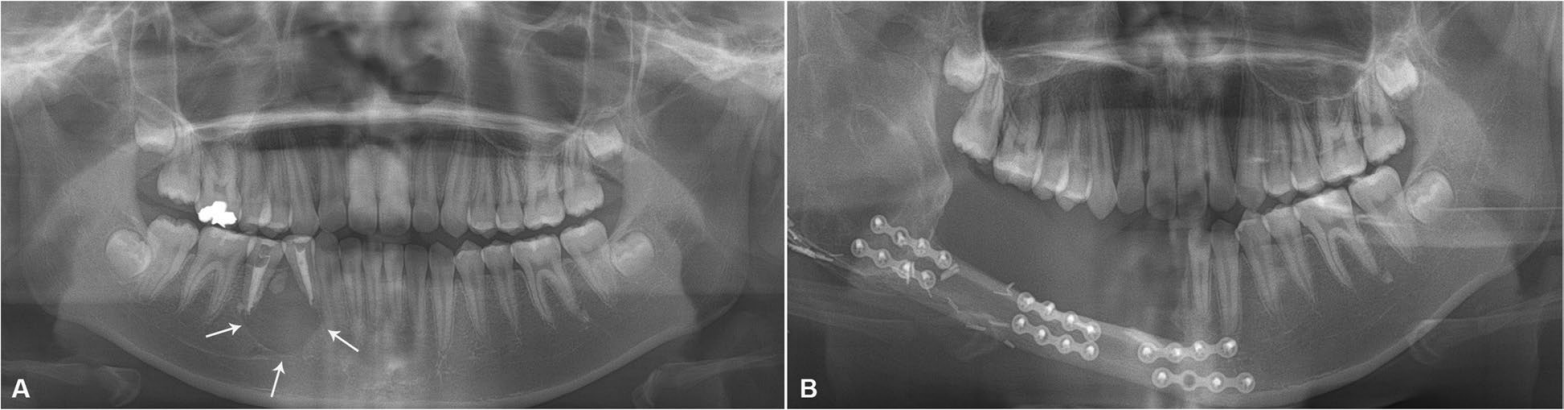
Panoramic view findings. **a** Two days before initial treatment. **b** One month after the end of concurrent chemo-radiation therapy

In this study, a new case was described under the approval of the Institutional Review Board of Seoul National University (S-D20200010), and we received the patient’s consent to participate. A 14-year-old female patient was referred from a local dental clinic to the Department of Oral and Maxillofacial Surgery at Seoul National University Dental Hospital, Seoul, Korea. She presented with painful swelling on the mandibular right premolar area and complained of intermittent bleeding when she brushed her teeth. She has no significant past medical history, and her family has no history of cancer. The panoramic view showed a radiolucent lesion with a well-defined border (Fig. 2a). Adjacent tooth displacement and external root resorption were noted. There was a radiopaque focus in the upper area of the lesion. The lesion was diagnosed as odontogenic cyst. Incisional biopsy or FNAC was not performed preoperatively. The cystic mass was enucleated with extraction of the right first premolar under local anesthesia.

## Results

### Study selection

A total of 235 articles were retrieved using the search string. Among them, 140 articles were selected and 98 articles were excluded as they did not report PIOC. After a full-text review, another 15 articles were excluded as they were not written in English. Additional three articles were obtained from the references in the retrieved reports. Another 114 articles were excluded as they did not report pediatric or adolescent cases. A total of 11 articles including 11 cases were found to satisfy inclusion criteria (Fig. 1) [12–22]. The clinical and radiologic characteristics of all 12 pediatric and adolescent cases including the present new case were summarized in Table 1. Treatment outcomes of 12 cases were summarized in Table 2.

**Table 1:** Clinical and radiological characteristics of 10 pediatric patients with primary intraosseous carcinoma in the jaw

Case	Year	Author	Country	Age	Sex	Location	Side	Symptom	Locularity	Density	Border	Tooth displacement	Root resorption
1	1966	Jones [12]	Ireland	4 years	F	Mandible	Right	Swelling	N/S	N/S	N/S	Yes	N/S
2	1973	Sirsat et al. [13]	India	16 years	M	Mandible	Left	Swelling	Not loculated	Mixed	Diffuse	Yes	N/S
3	2002	Gulbranson et al. [14]	America	16 months	F	Mandible	Right	Swelling	Unilocular	Radiolucency	Cortical thinning	N/S	N/S
4	2004	Punnya et al. [15]	India	18 years	F	Maxilla	Right	Swelling	N/S	Radiolucency, multiple radiopaque foci	Well-defined	N/S	Yes
5	2006	Chaisuparat et al. [16]	America	18 years	F	Maxilla	Right	Swelling	Unilocular	Radiolucency,	Well-defined, non-corticated	N/S	N/S
6	2006	Aboul-hosn et al. [17]	Spain	18 years	M	Maxilla	Right	No symptom	N/S	N/S	N/S	N/S	N/S
7	2008	Charles et al. [18]	Canada	5 years	F	Mandible	Right	Swelling	Unilocular	Radiolucency, enlarged crypt,	Well-defined, corticated	Yes	Yes
8	2010	Sengupta et al. [19]	India	16 years	M	Mandible	Right	Swelling, pain	Unilocular	Radiolucency	Well-defined, corticated	N/S	Yes
9	2015	Gay-Escoda et al. [20]	Spain	18 years	M	Maxilla	Left	Swelling, pain	Unilocular	Radiolucency	Well-defined, corticated	No	Yes
10	2016	Boni et al. [21]	Italy	14 years	M	Maxilla	N/S	Swelling	N/S	N/S	N/S	No	No
11	2018	Nokovitch et al. [22]	France	15 years	F	Mandible	Right	Swelling, pain, intermittent bleeding	Unilocular	Radiolucency, supernumerary tooth	Lingual cortex lysis	N/S	Yes
12	2020	The present study	Korea	14 years	F	Mandible	Right	Swelling, pain, intermittent bleeding	Unilocular	Radiolucency, a radiopaque focus	Well-defined, corticated	Yes	Yes

*N/S* not specified

**Table 2:** Treatment outcomes of 10 pediatric patients with primary intraosseous carcinoma in the jaw

Case	Initial diagnosis	Initial treatment	Confirmed diagnosis	Definitive treatment	Cervical metastasis	Local recurrence	Salvage treatment	Follow-up duration	Survival status at the last F/U
1	N/S	N/S	N/S	Excision	Yes ^a^	Yes	Radical excision/SND, RT ^a^	27 months	Alive
2	N/S	N/S	N/S	Excision	Yes ^b^	Yes	Mandibulectomy, SND	8 months	Dead
3	Dentigerous cyst	Incisional biopsy	Carcinoma	Mandibulectomy, SND	No	No	No	8 months	Alive
4	Odontogenic cyst	FNAC	Odontogenic cyst	Enucleation	N/S	No	No	16 months	Alive
5	N/S	N/S	N/S	Maxillectomy, RT	No	No	No	44 months	Alive
6	Follicular cyst	Enucleation with extraction	PIOC	Maxillectomy, reconstruction with iliac crest	No	No	No	10 years	Alive
7	More aggressive lesion than odontogenic cyst	Incisional biopsy with extraction	PIOC	Mandibulectomy, mRND, reconstruction with plate, RT	Yes	No	No	7 years	Alive
8	OKC	Enucleation with extraction	PIOC	Refer	N/S	N/S	N/S	N/S	N/S
9	Dentigerous cyst	Enucleation with extraction	PIOC	Maxillectomy, reconstruction with plate and iliac crest	No	No	No	6 years	Alive
10	N/S	N/S	N/S	Maxillectomy	N/S	No	No	5 years	Alive
11	OKC	Incisional biopsy	OKC	Enucleation with extraction	No	No	No	18 months	Alive
12	Odontogenic cyst	Enucleation with extraction	PIOC	Mandibulectomy, SND, reconstruction with FFF	No	Yes	CCRT	9 months ^c^	Alive

*F/U* follow-up, *N/S* not specified, *OKC* odontogenic keratocyst, *FNAC* fine needle aspiration cytology, *PIOC* primary intraosseous carcinoma, *SND* selective neck dissection, *mRND* modified radical neck dissection, *RT* radiation therapy, *CCRT* concurrent chemo-radiation therapy, *FFF* fibular free flap

^a^At second recurrence

^b^At recurrence

^c^Recurred but lost to follow-up

### Clinical and radiologic findings

Ages ranged from 4 to 18 years. Most of the patients were from 14 to 18 years except two patients aged 4 and 5 years. Seven patients were females and five patients were males with a female to male ratio of 1.4:1. PIOC was found in the mandible in seven cases and in the maxilla in five cases. There were nine cases in the right side and two cases in the left side. Swelling was observed in eleven patients. Four patients complained of pain. Intermittent bleeding accompanied two of four patients.

The lesions were unilocular in seven cases and not loculated in one case. Eight cases were radiolucent and one case was mixed radiolucent and radiopaque. A radiopaque focus or multiple foci were observed in two cases, accompanied with radiolucency. The borders of the lesions were well-defined in six cases and corticated borders were found in four of six cases. Tooth displacement was observed in four cases and there was no tooth displacement in two cases. Six cases showed root resorption, one case did not, and the others were unspecified.

### Treatment outcomes

The initial diagnosis for seven cases was odontogenic cyst. Two of seven cases were diagnosed as dentigerous cyst and diagnosis of another two cases was odontogenic keratocyst. Incisional biopsy was performed in three cases and fine-needle aspiration cytology (FNAC) was investigated in one case. All the patients underwent surgical treatment. Six patients received surgical enucleation with the diagnosis of odontogenic cyst. Nine patients underwent extensive resection as definitive or salvage treatment. Five patients received neck dissection surgery. Three cases showed cervical metastasis, six cases did not, and the others were unspecified. Post-operative radiation therapy was performed in three cases. During the follow-up period, local recurrence occurred in three patients. Two patients underwent extensive resection with neck dissection for the local recurrence. After the surgery, one patient was alive but the other patient was dead.

### Progress for the 14-year-old patient

**Fig. 3:**
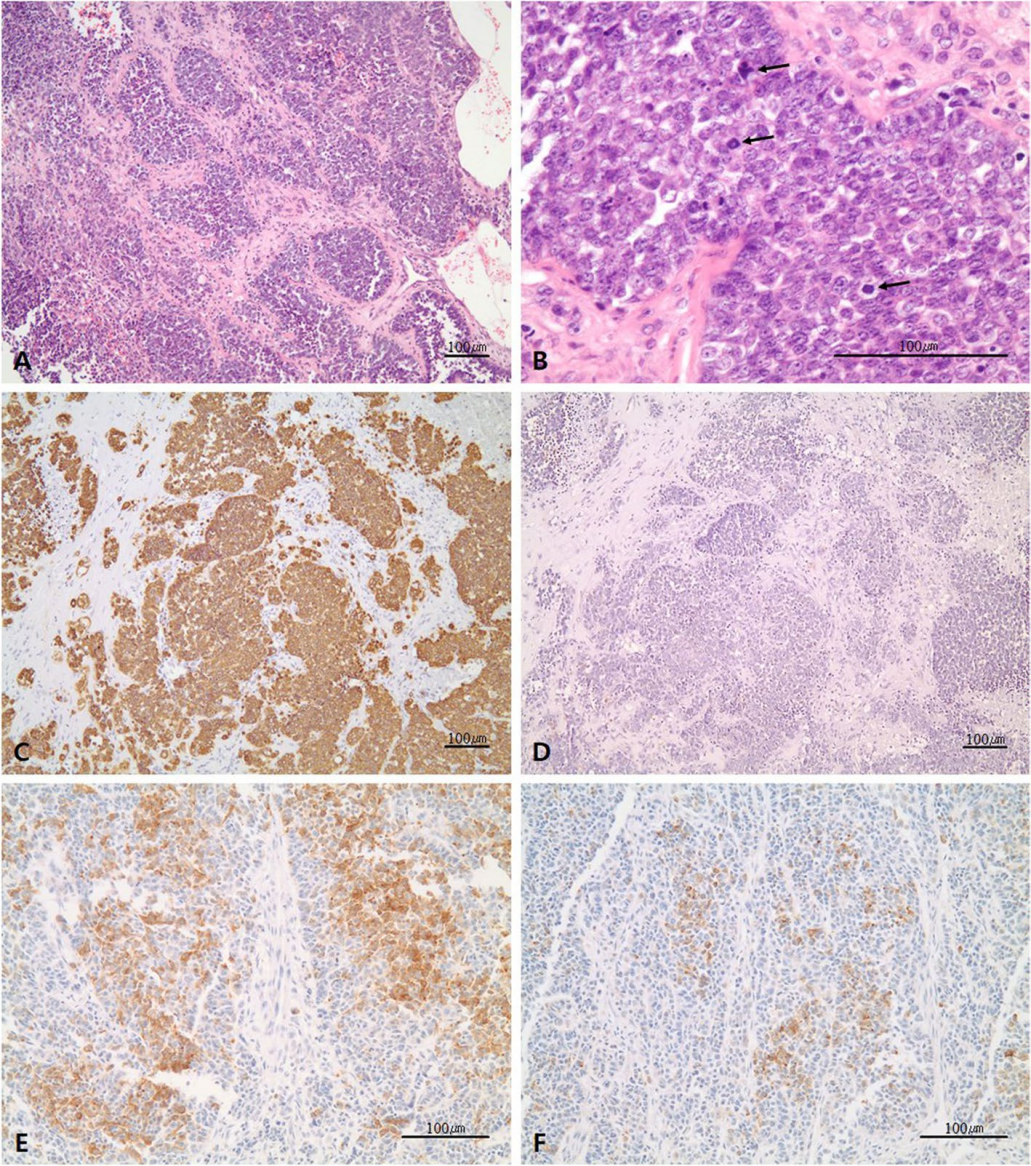
Representative histopathological features. **a** Solid nests or sheets of tumor cells with high cellularity. **b** Round monotonous tumor cells showing high N/C ratio and abnormal mitoses (indicated by the arrows). **c** Diffuse strong positivity for CK-pan. **d** Negative expression of CD99. **e** Focal immunoreactivity for synaptophysin. **f** Focal immunoreactivity for chromogranin A

After enucleation surgery, histopathology of the surgical specimen revealed a highly cellular tumor growing in solid nests or sheets (Fig. 3a). Microscopic examination showed round monotonous-shaped tumor cells with a high nucleus-cytoplasm ratio and abnormal mitoses (Fig. 3b). On immunohistochemical staining, tumor cells showed diffuse positivity for CK-pan (Fig. 3c), but focal for CK-7 and vimentin. Moreover, cells were 40–60% positive for Ki-67. However, immunoreactivities for CD99, desmin, S-100 protein, and NSE were all negative (Fig. 3d). In addition, immunoreactivities for neuroendocrine markers including synaptophysin and chromogranin A were not diffusely, but focally, positive (Fig. 3e, f). Since peripheral palisading or reverse nuclear polarity was not observed, ameloblastic carcinoma was ruled out. In addition, neuroendocrine carcinoma was ruled out based on the immunohistochemical results, considering that neuroendocrine carcinoma generally shows diffuse and strong expression of neuroendocrine markers [28–30]. A final diagnosis was consistent with PIOC. The patient was referred to the cancer and reconstruction team in the same department.

**Fig. 4:**
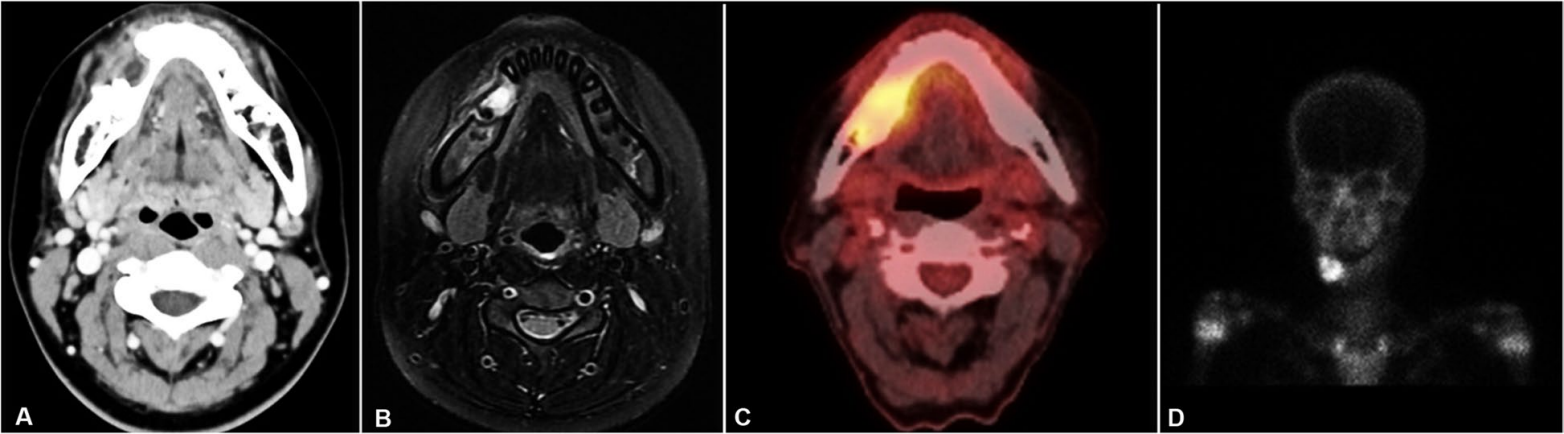
Pre-operative imaging. **a** Enhanced CT showing periosteal reaction at the buccal side. **b** MRI showing diffuse enhancement of soft tissue at the adjacent buccal area. **c** PET-CT showing a soft tissue lesion at the right premolar lesion. **d** Bone scintigraphy showing increased uptake in the right mandible

Enhanced CT, magnetic resonance imaging (MRI), positron emission tomography-CT (PET-CT), bone scintigraphy, and neck ultrasonography were performed. On enhanced CT, a periosteal reaction was observed on the buccal side (Fig. 4a). On MRI, diffuse enhancement of the soft tissue was observed at the adjacent buccal area (Fig. 4b). There were no significant lymph nodes. In PET-CT, a soft tissue lesion on the right premolar area and a borderline-sized lymph node at the right level IB were observed (Fig. 4c). There was no distant metastasis. In bone scintigraphy, increased uptake in the right mandible was observed (Fig. 4d). On neck ultrasonography, no significant cervical lymph node enlargement was observed. Hand-wrist radiograph and lower extremity angiography were performed for reconstruction surgery with the fibular free flap. In hand-wrist radiograph, that patient’s skeletal maturation was estimated as 15–16 years old. On leg angiography, anterior tibial, posterior tibial, and peroneal arteries were intact.

**Fig. 5:**
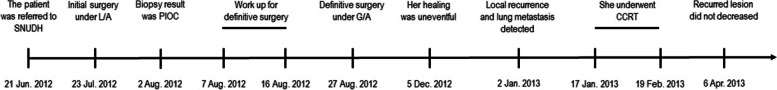
Time table with chronologic events. *SNUDH* Seoul National University, *L/A* local anesthesia, *PIOC* primary intraosseous carcinoma, *G/A* general anesthesia, *CCRT* concurrent chemo-radiation therapy

**Fig. 6:**
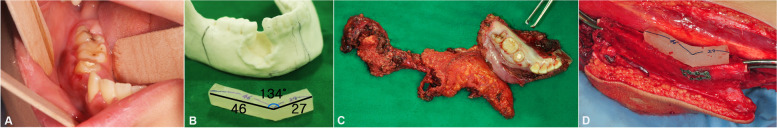
Surgical procedures. **a** Abnormal healing 1 month after enucleation surgery and one day before definitive surgery. **b** The 73-mm resin stent for reconstruction with fibula-free flap. **c** En bloc resection of the right mandible and neck mass (right level I, II, III). **d** Intraoperative fibular contouring using the stent

Definitive surgery was performed approximately 1 month after the enucleation surgery. The biopsy result was confirmed about 2 weeks after the enucleation surgery, and it took about 2 weeks for cancer work-up with consideration of reconstruction surgery (Fig. 5). The patient showed abnormal healing (Fig. 6a). Surgical resection and reconstruction with a fibula-free flap were prepared. The required bone length was 73 mm, and a resin stent was prepared (Fig. 6b). She underwent segmental mandibulectomy [31–33] from the right retromolar area to the left central incisor and selective neck dissection (right levels I, II, and III) under general anesthesia. The mandibular and neck masses were removed en bloc (Fig. 6c) with simultaneous reconstruction using the microvascular fibula-free flap (Fig. 6d).

The surgical resection margin was clear, and no metastatic cervical lymph nodes were found in the dissected mass. Perineural or vascular invasion was not seen. However, there was involvement of the underlying bone. The patient’s healing was uneventful. However, local recurrence and lung metastasis occurred 4 months after surgery. She underwent CCRT for 1 month; however, a recurred lesion on the right mandibular ramus did not decrease in size (Fig. 2b). She was lost to follow-up after her visit 1 month after the end of CCRT.

## Discussion

PIOC is more common in adult males [3, 5–11]; however, seven of the total 12 pediatric and adolescent cases were female. PIOC occurs more often in the mandible than in the maxilla [3, 5–11], seen in seven of the pediatric and adolescent patients. PIOC showed a predilection for the right side, observed in nine of the pediatric and adolescent patients. The most common symptom was swelling, followed by pain [3, 5, 7]. The symptoms of the pediatric and adolescent patients were consistent.

PIOC can be misdiagnosed as odontogenic cyst because it occasionally presents with a well-defined border in panoramic view or CT [23–26]. The initial diagnosis as odontogenic cyst in the present study was performed based on the clinical signs and the panoramic view. The border of the lesion was well-defined and corticated. The mesial border was somewhat blurred with sclerotic change. A radiopaque focus with a well-defined border was observed in the lesion, which could be suspected as calcifying odontogenic cyst [34, 35]. However, further imaging such as CT should be carried out to identify aggressive pattern or expansion of the lesion even if odontogenic cyst was suspected. Differential diagnosis of PIOC from odontogenic cyst is important because the surgical approach is different. Kaffe et al. [23] reported that 61% of PIOC cases presented as a unilocular radiolucent lesion. In our review of pediatric and adolescent cases, seven of 12 cases showed unilocular radiolucency. Radiologic borders that were defined but non-corticated were reported to occur in 57% of the PIOC cases and the remaining 43% had diffuse borders. In cases with poorly defined borders, such as those with diffuse margins, the lesions could be unambiguously distinguished from odontogenic cyst. However, since cases with well-defined borders can be misdiagnosed as odontogenic cyst, a differential diagnosis should be thoroughly considered. According to Kaffe et al. [23], a defined but non-corticated border could be a useful feature for differential diagnosis. A well-defined border was observed in six of 12 pediatric and adolescent PIOC patients (Table 1). However, a well-defined but non-corticated border was observed in only one of six cases where the borders of the lesions could be identified on the radiographic images shown in the papers. Tooth displacement and root resorption should be considered other radiologic features since PIOC tends to grow too rapidly to produce such features [7, 21, 23, 36]. However, four of the pediatric and adolescent cases in our review showed tooth displacement and six showed root resorption. It was peculiar that these features occurred in pediatric and adolescent patients. Although tooth displacement and root resorption are features for slowly growing lesions such as odontogenic cysts, these features should be considered for differential diagnosis of PIOC in pediatric and adolescent populations. As a rare finding, radiopaque foci were observed in this case and in Punnya et al.’s adolescent case [15]. Although PIOC usually presents as an osteolytic lesion, small radiopaque foci due to calcification or periosteal reaction can be observed, albeit rarely [10, 37–39].

Among the 12 pediatric and adolescent cases we reviewed, the initial diagnosis for five was odontogenic cyst. Huang et al. [7] reported that this diagnostic delay did not show any statistically significant prognostic difference. However, Naruse et al. [36] reported that preoperative dental procedures might be potential prognostic factors and suggested that no intervention before definitive diagnosis could achieve a better prognosis. Therefore, incisional biopsy with obtaining multiple specimens is necessary to rule out an underlying carcinoma [8, 9, 14, 18]. Regardless of patient age, biopsy should be considered for any lesion with any of the unusual radiographic presentations mentioned above. The pediatric patient reported by Charles et al. [18] was accurately diagnosed by biopsy and had the longest follow-up period without recurrence. A biopsy was not considered for definitive diagnosis in the present case although there were atypical radiographic findings. Local recurrence occurred 5 months after the initial operation, that is, 4 months after the definitive surgery. Meanwhile, the efficacy of FNAC for diagnosis of intraosseous jaw pathology has not been well-established [40]. Radiolucent jaw lesions are occasionally amenable to FNAC due to thinning of the bony cortex [41]. FNAC can be useful to differentiate benign from malignant tumors [42, 43]. Therefore, the aspiration could be helpful in the present case. Among 12 cases, FNAC was performed in Punnya et al.’s case [15]. The cytopathology showed minimally pleomorphic and hyperchromatic cells suggesting an epithelial lesion of odontogenic origin. The provisional diagnosis of the lesion was odontogenic cyst. Likewise, in the 50-year-old male patient in Thomas et al.’s study [5], FNAC was inconclusive. However, in the 35-year-old male patient in the same study, FNAC was suggestive of malignant neoplastic growth. Moreover, in the 70-year-old male patient in Lugakingira et al.’s study [8], FNAC suggested a solid tumor. Therefore, FNAC could be used as an adjunct method to the incisional biopsy for definitive diagnosis [44].

The primary treatment for PIOC is surgical resection [3, 8, 11, 45]. In the present case, because simultaneous reconstruction was necessary, hand-wrist radiography was analyzed for assessment of growth potential. Her skeletal age was assessed as 15–16 years old. Previous research has concluded that the face matures between 12 and 15 years in males and 2 years earlier in females [46, 47]. The vascularized fibular free flap is a reliable option for mandibular reconstruction, even in pediatric and adolescent patients [48, 49]. Therefore, the fibular free flap was employed for this 14-year-old female patient.

Recent reviews reported the rate of cervical lymph node metastasis to be 12.8% [3] and 70.1% [11]. In our case review, three of the 12 pediatric and adolescent cases showed cervical metastasis. Wenguang et al. [11] reported nodal status to be a significantly poor prognostic factor for survival. However, de Morais et al. [3] reported that lymph node metastasis was not statistically associated with survival. Although these outcomes conflict, it seems reasonable that neck dissection be considered among the surgical procedures for PIOC. In the present case, there were no metastatic lymph nodes on enhanced CT or MRI. However, since there were borderline-sized lymph nodes at the right level IB and supraomohyoud neck dissection was a reliable procedure in patients who has clinically negative or limited cervical metastasis, selective neck dissection was performed [50–52].

In recent literature, de Morais et al. [3] reported a local recurrence rate of 22.1% and Ye et al. [4] reported a local recurrence rate of 24.1%. In this study, local recurrence occurred in the mandible of three pediatric and adolescent patients, including the present case, a rate of 25% although the total cases were only 12. In one report, a 4-year-old female patient suffered recurrence 5 months after excision and underwent additional radical excision [3]. However, 10 months later, recurrence recurred, the lesion was removed, and the area was irradiated. She was alive after 1 year of follow-up. In another case, a 16-year-old male patient suffered recurrence 2 months after excision and underwent total mandibulectomy after one month. However, he died 2 months after the surgery [13]. The 14-year-old female patient in the present study suffered recurrence 4 months after definitive surgery. She underwent CCRT for 1 month, but the recurred lesion did not decrease in size. According to de Morais et al. [3] and Ye et al. [4], local recurrence is a significant prognostic factor for survival. Likewise, pediatric and adolescent PIOC cases with local recurrence showed poor prognosis. The 5-year survival rate has been reported as 44.6% [3] and 53.2% [4]. However, the 5-year survival rate of the pediatric and adolescent patients could not be evaluated because of the rarity of the cases and relatively short follow-up periods. The follow-up duration was shorter than 2 years in five cases among 12 pediatric and adolescent patients. Among the cases of death, most patients died before 2 years after initial diagnosis [3].

Because PIOC has a poor prognosis, accurate diagnosis and adequate surgical procedures are important. Continuous updates are required to analyze the pathophysiologic mechanism of PIOC, and a recent approach such as genetic analysis [53] could contribute to understanding the pathophysiology of PIOC.

## Conclusions

PIOC is a rare malignant odontogenic tumor that can be misdiagnosed as odontogenic cyst because it occasionally presents with a well-defined border on radiography. According to the literature review, 12 pediatric and adolescent PIOC cases have been reported, seven of which were initially diagnosed as odontogenic cyst. Atypically, tooth displacement and root resorption were observed in one-third and one-half of the pediatric and adolescent cases, respectively. Three-dimensional imaging modalities and incisional biopsy with multiple specimens are necessary to rule out PIOC in the cases with atypical radiographic findings. FNAC can prevent mismanagement of the disease with a poor prognosis. Local recurrence seemed to be a significant prognostic factor for survival in pediatric and adolescent PIOC cases consistent with adult cases. PIOC should be differentially diagnosed from odontogenic cyst even in pediatric and adolescent populations.

## Data Availability

The datasets used and/or analyzed during the current study are available from the corresponding author upon reasonable request.

## Abbreviations

PIOC: Primary intraosseous carcinoma
WHO: World health organization
CT: computed tomography
PRISMA: Preferred Reporting Items for Systematic Reviews and Meta-Analyses
FNAC: Fine-needle aspiration cytology
MRI: Magnetic resonance imaging
PET-CT: Positron emission tomography-computed cosmography
CCRT: Concurrent chemo-radiation therapy

## References

[1] Eversole LR, Siar CH, van der Waal I, Barnes L, Eveson JW, Reichart P, Sidransky D (2005). Primary intraosseous squamous cell carcinomas. Pathology and genetics of head and neck tumours.

[2] Wright JM, Vered M (2017). Update from the 4th edition of the World Health Organization classification of head and neck tumours: odontogenic and maxillofacial bone tumors. Head Neck Pathol.

[3] de Morais EF, Carlan LM, de Farias Morais HG, Pinheiro JC, Martins HDD, Barboza CAG, et al. Primary intraosseous squamous cell carcinoma involving the jaw bones: a systematic review and update. Head Neck Pathol. 2020. 10.1007/s12105-020-01234-z PMC8134565 33044723

[4] Ye P, Wei T, Gao Y, Zhang W, Peng X (2021). Primary intraosseous squamous cell carcinoma arising from an odontogenic keratocyst: case series and literature review. Med Oral Patol Oral Cir Bucal.

[5] Thomas G, Pandey M, Mathew A, Abraham EK, Francis A, Somanathan T (2001). Primary intraosseous carcinoma of the jaw: pooled analysis of world literature and report of two new cases. Int J Oral Maxillofac Surg.

[6] Zwetyenga N, Pinsolle J, Rivel J, Majoufre-Lefebvre C, Faucher A, Pinsolle V (2001). Primary intraosseous carcinoma of the jaws. Arch Otolaryngol Head Neck Surg.

[7] Huang JW, Luo HY, Li Q, Li TJ (2009). Primary intraosseous squamous cell carcinoma of the jaws. Clinicopathologic presentation and prognostic factors. Arch Pathol Lab Med.

[8] Lugakingira M, Pytynia K, Kolokythas A, Miloro M (2010). Primary intraosseous carcinoma of the mandible: case report and review of the literature. J Oral Maxillofac Surg.

[9] Bodner L, Manor E, Shear M, van der Waal I (2011). Primary intraosseous squamous cell carcinoma arising in an odontogenic cyst: a clinicopathologic analysis of 116 reported cases. J Oral Pathol Med.

[10] Matsuzaki H, Katase N, Matsumura T, Hara M, Yanagi Y, Nagatsuka H (2012). Solid-type primary intraosseous squamous cell carcinoma of the mandible: a case report with histopathological and imaging features. Oral Surg Oral Med Oral Pathol Oral Radiol.

[11] Wenguang X, Hao S, Xiaofeng Q, Zhiyong W, Yufeng W, Qingang H (2016). Prognostic factors of primary intraosseous squamous cell carcinoma (PIOSCC): a retrospective review. PLoS One.

[12] Jones JH (1966). Soft tissue oral tumours in children: their structure, histogenesis and behaviour. Proc R Soc Med.

[13] Sirsat MV, Sampat MB, Shrikhande SS (1973). Primary intra-alveolar squamous-cell carcinoma of the mandible. Report of a case. Oral Surg Oral Med Oral Pathol.

[14] Gulbranson SH, Wolfrey JD, Raines JM, McNally BP (2002). Squamous cell carcinoma arising in a dentigerous cyst in a 16-month-old girl. Otolaryngol Head Neck Surg.

[15] Punnya A, Kumar GS, Rekha K, Vandana R (2004). Primary intraosseous odontogenic carcinoma with osteoid/dentinoid formation. J Oral Pathol Med.

[16] Chaisuparat R, Coletti D, Kolokythas A, Ord RA, Nikitakis NG (2006). Primary intraosseous odontogenic carcinoma arising in an odontogenic cyst or de novo: a clinicopathologic study of six new cases. Oral Surg Oral Med Oral Pathol Oral Radiol Endod.

[17] Aboul-hosn Centenero S, Mari-Roig A, Piulachs-Clapera P, Juarez-Escalona I, Monner-Dieguez A, Diaz-Carandell A (2006). Primary intraosseous carcinoma and odontogenic cyst. Three new cases and review of the literature. Med Oral Patol Oral Cir Bucal.

[18] Charles M, Barr T, Leong I, Ngan BY, Forte V, Sandor GK (2008). Primary intraosseous malignancy originating in an odontogenic cyst in a young child. J Oral Maxillofac Surg.

[19] Sengupta S, Vij H, Vij R (2010). Primary intraosseous carcinoma of the mandible: a report of two cases. J Oral Maxillofac Pathol.

[20] Gay-Escoda C, Camps-Font O, Lopez-Ramirez M, Vidal-Bel A (2015). Primary intraosseous squamous cell carcinoma arising in dentigerous cyst: report of 2 cases and review of the literature. J Clin Exp Dent.

[21] Boni P, Sozzi D, Novelli G, Pagni F, Valente G, Bozzetti A (2016). Primary intraosseous squamous cell carcinoma of the jaws: 6 new cases, experience, and literature comparison. J Oral Maxillofac Surg.

[22] Nokovitch L, Bodard AG, Corradini N, Crozes C, Guyennon A, Deneuve S (2018). Pediatric case of squamous cell carcinoma arising from a keratocystic odontogenic tumor. Int J Pediatr Otorhinolaryngol.

[23] Kaffe I, Ardekian L, Peled M, Machtey E, Laufer D (1998). Radiological features of primary intra-osseous carcinoma of the jaws. Analysis of the literature and report of a new case. Dentomaxillofac Radiol.

[24] Scheer M, Koch AM, Drebber U, Kubler AC (2004). Primary intraosseous carcinoma of the jaws arising from an odontogenic cyst--a case report. J Craniomaxillofac Surg.

[25] Choi YJ, Oh SH, Kang JH, Choi HY, Kim GT, Yu JJ (2012). Primary intraosseous squamous cell carcinoma mimicking periapical disease: a case report. Imaging Sci Dent.

[26] Abdelkarim AZ, Elzayat AM, Syed AZ, Lozanoff S (2019). Delayed diagnosis of a primary intraosseous squamous cell carcinoma: a case report. Imaging Sci Dent.

[27] Moher D, Liberati A, Tetzlaff J, Altman DG, Group P (2009). Preferred reporting items for systematic reviews and meta-analyses: the PRISMA statement. J Clin Epidemiol.

[28] Sugawara C, Takahashi A, Kawano F, Kudoh T, Yamada A, Ishimaru N (2015). Neuroendocrine tumor in the mandible: a case report with imaging and histopathologic findings. Oral Surg Oral Med Oral Pathol Oral Radiol.

[29] Said-Al-Naief N, Sciandra K, Gnepp DR (2013). Moderately differentiated neuroendocrine carcinoma (atypical carcinoid) of the parotid gland: report of three cases with contemporary review of salivary neuroendocrine carcinomas. Head Neck Pathol.

[30] Terada T (2013). Small cell carcinoma of the oral cavity (cheek mucosa): a case report with an immunohistochemical and molecular genetic analysis. Int J Clin Exp Pathol.

[31] Wax MK, Bascom DA, Myers LL (2002). Marginal mandibulectomy vs segmental mandibulectomy: indications and controversies. Arch Otolaryngol Head Neck Surg.

[32] Misra S, Chaturvedi A, Misra NC (2008). Management of gingivobuccal complex cancer. Ann R Coll Surg Engl.

[33] Gou L, Yang W, Qiao X, Ye L, Yan K, Li L (2018). Marginal or segmental mandibulectomy: treatment modality selection for oral cancer: a systematic review and meta-analysis. Int J Oral Maxillofac Surg.

[34] Buchner A (1991). The central (intraosseous) calcifying odontogenic cyst: an analysis of 215 cases. J Oral Maxillofac Surg.

[35] Iida S, Fukuda Y, Ueda T, Aikawa T, Arizpe JE, Okura M (2006). Calcifying odontogenic cyst: radiologic findings in 11 cases. Oral Surg Oral Med Oral Pathol Oral Radiol Endod.

[36] Naruse T, Yanamoto S, Sakamoto Y, Ikeda T, Yamada SI, Umeda M (2016). Clinicopathological study of primary intraosseous squamous cell carcinoma of the jaw and a review of the literature. J Oral Maxillofac Surg.

[37] Bennett JH, Jones J, Speight PM (1993). Odontogenic squamous cell carcinoma with osseous metaplasia. J Oral Pathol Med.

[38] Ide F, Shimoyama T, Horie N, Kaneko T (1999). Primary intraosseous carcinoma of the mandible with probable origin from reduced enamel epithelium. J Oral Pathol Med.

[39] Lopes Dias J, Borges A, Lima RR (2016). Primary intraosseous squamous cell carcinoma of the mandible: a case with atypical imaging features. BJR Case Rep.

[40] August M, Faquin WC, Ferraro NF, Kaban LB (1999). Fine-needle aspiration biopsy of intraosseous jaw lesions. J Oral Maxillofac Surg.

[41] Singh S, Garg N, Gupta S, Marwah N, Kalra R, Singh V (2011). Fine needle aspiration cytology in lesions of oral and maxillofacial region: Diagnostic pitfalls. J Cytol.

[42] Goyal S, Sharma S, Kotru M, Gupta N (2015). Role of FNAC in the diagnosis of intraosseous jaw lesions. Med Oral Patol Oral Cir Bucal.

[43] Platt JC, Rodgers SF, Davidson D, Nelson CL (1993). Fine-needle aspiration biopsy in oral and maxillofacial surgery. Oral Surg Oral Med Oral Pathol.

[44] Baykul T, Colok G, Gunhan O (2010). The value of aspiration cytology in cystic lesions of the maxillofacial region. Eur J Dent.

[45] Woolgar JA, Triantafyllou A, Ferlito A, Devaney KO, Lewis JS, Rinaldo A (2013). Intraosseous carcinoma of the jaws: a clinicopathologic review. Part III: primary intraosseous squamous cell carcinoma. Head Neck.

[46] Farkas LG, Posnick JC, Hreczko TM (1992). Growth patterns of the face: a morphometric study. Cleft Palate Craniofac J.

[47] Li JS, Chen WL, Huang ZQ, Zhang DM (2009). Pediatric mandibular reconstruction after benign tumor ablation using a vascularized fibular flap. J Craniofac Surg.

[48] Guo L, Ferraro NF, Padwa BL, Kaban LB, Upton J (2008). Vascularized fibular graft for pediatric mandibular reconstruction. Plast Reconstr Surg.

[49] Benoit MM, Vargas SO, Bhattacharyya N, McGill TA, Robson CD, Ferraro N (2013). The presentation and management of mandibular tumors in the pediatric population. Laryngoscope..

[50] Medina JE, Byers RM (1989). Supraomohyoid neck dissection: rationale, indications, and surgical technique. Head Neck.

[51] Spiro RH, Morgan GJ, Strong EW, Shah JP (1996). Supraomohyoid neck dissection. Am J Surg.

[52] Chepeha DB, Hoff PT, Taylor RJ, Bradford CR, Teknos TN, Esclamado RM (2002). Selective neck dissection for the treatment of neck metastasis from squamous cell carcinoma of the head and neck. Laryngoscope..

[53] Yukimori A, Tsuchiya M, Wada A, Michi Y, Kayamori K, Sakamoto K (2020). Genetic and histopathological analysis of a case of primary intraosseous carcinoma, NOS with features of both ameloblastic carcinoma and squamous cell carcinoma. World J Surg Oncol.

